# Harmonizing Data Visualizations on Child Health and Well-Being to Strengthen Advocacy and Monitoring Efforts

**DOI:** 10.9745/GHSP-D-23-00183

**Published:** 2023-12-22

**Authors:** Jennifer Harris Requejo, Kathleen Strong, Frances Aboud, Ambrose Agweyu, Sk Masum Billah, Maureen Black, Cynthia Boschi-Pinto, Sayaka Horiuchi, Zeina Jamaluddine, Marzia Lazzerini, Abdoulaye Maiga, Melinda Munos, Joanna Schellenberg, Ralf Weigel, Emma Sacks, for the Child Health Accountability Technical Advisory Group

**Affiliations:** a The World Bank Group, Global Financing Facility, Washington, DC, USA.; b Johns Hopkins Bloomberg School of Public Health, Baltimore, MD, USA.; c World Health Organization, Geneva, Switzerland.; d McGill University, Montreal, Canada.; e Health Services Unit, KEMRI-Wellcome Trust Research Programme, Nairobi, Kenya.; f Maternal and Child Health Division, icddr,b, Dhaka, Bangladesh.; g Department of Pediatrics and Department of Epidemiology and Public Health, University of Maryland School of Medicine, Baltimore, MD, USA; RTI International, Research Triangle Park, NC, USA.; h University Federal Fluminense, Rio de Janeiro, Brazil.; i Center for Birth Cohort Studies, University of Yamanashi, Yamanashi, Japan.; j London School of Hygiene and Tropical Medicine, London, United Kingdom.; k American University of Beirut, Beirut, Lebanon.; l Institute for Maternal and Child Health IRCCS Burlo Garofolo, Trieste, Italy.; m Witten/Herdecke University, Witten, Germany.; n Consultant, Child Health Accountability Tracking Technical Advisory Group.

## Abstract

Data visualization tools on child health have improved data accessibility but caused confusion over indicator data sources and which tools to use for specific purposes. We propose principles for generating future tools that can effectively trigger action and accountability for children everywhere.

## Key Messages

- Although the substantial increase in data visualization tools on child health and well-being has made data more accessible and facilitated evidence-based decision-making, the increase has contributed to confusion over which tools to use for specific purposes and how they complement each other.
- We summarize the events that triggered increased production of data visualization tools on child health and well-being, discuss the benefits and challenges resulting from this increase, and propose principles for producing future tools, with an emphasis on tools for global monitoring, so that they most effectively spark action and accountability for children.
- The 6 principles include: (1) cocreate data visualization tools across organizations; (2) align donors on requests for new child health data visualization tools; (3) define the purpose, target audience, and added value of new tools at the outset; (4) develop tools through inclusive engagement of all key stakeholders; (5) identify a lead institution or individual to manage iterations of the tool and build consensus; and (6) select indicators that adhere to standard definitions, recommended data sources, and are available through existing data collection platforms.

## INTRODUCTION

In the past few decades, there has been a proliferation of country profiles and interactive web-based dashboards that graphically present data on child health and well-being. These tools are designed to make data more accessible to a wide range of audiences, facilitate simple analyses and interpretation of data, and foster greater uptake of data for decision-making as well as for accountability.^1^^,^^2^ However, the growing volume of data visualization tools has created confusion over which ones to use for specific purposes (e.g., global monitoring, national and subnational monitoring, program-specific monitoring, and advocacy and accountability), how they complement each other (i.e., how to use global dashboards in combination with more detailed subnational data for planning and resource allocation purposes), and where the data come from. We summarize key historical events that fostered the surge in data visualization tools on child health and well-being, discuss the benefits and challenges that have resulted from this surge, and present a proposed set of principles for producing future tools for global monitoring and cross-country comparisons.

## THE RISE OF STATISTICAL PROFILES ON CHILD HEALTH AND WELL-BEING: A BRIEF HISTORY

The increase in data visualization tools on child health is linked to advances in data analytics, improvement in data availability, and the emergence of global accountability frameworks that include goals and associated targets. Assessing progress toward these goals and targets necessitates good measurement, regular monitoring, and effective communication about the indicators used. The World Declaration on the Survival, Protection, and Development of Children and the associated plan of action for its implementation (Declaration)^3^ adopted by the World Summit for Children in New York in September 1990 was the first major framework with goals and targets for children. This framework was consistent with the Convention on the Rights of the Child adopted the year before by the United Nations General Assembly^4^ and the ethos of the Child Survival Revolution launched in 1982.^5^ The framework included 27 goals covering child survival, child development and protection, education, and nutrition. In recognition of the importance of maternal health and social determinants for child health and well-being, goals were also included on women's health, water and sanitation, and poverty.^3^

The establishment of these goals triggered demand for rigorous annual reviews of progress toward their achievement based on comparable, reliable data and simple visualizations that policymakers could readily understand. In response, agencies with a remit on child health, like UNICEF and the World Health Organization (WHO), began strengthening their monitoring and evaluation capacities and improving the display of data from their publicly accessible global databases. In the mid-1980s, the U.S. Agency for International Development-supported Demographic and Health Surveys (DHS)^6^ were rolled out to systematically measure health outcomes for women and children and were followed by the introduction of the UNICEF-supported Multiple Indicator Cluster Surveys in the early 1990s.^7^

The establishment of goals for child survival and development triggered demand for rigorous annual reviews of progress toward their achievement based on comparable, reliable data.

The mid- and end-decade evaluations of the 1990 Declaration resulted in a call for reaffirmation of the commitments made to children, increased investments in social services for all, and greater economic and social empowerment of the poor.^8^^,^^9^ These aspirations were reflected in the outcomes of the September 2000 United Nations Millennium Summit when the United Nations Millennium Declaration was adopted with a set of time-bound goals and targets that placed maternal and child survival at their core.^10^ Two years later, a special session of the General Assembly was held to review progress on the Declaration and renew global commitment to children's rights. The result was adoption of the World Fit for Children resolution with a list of goals, targets, and strategies for improving children's lives.^8^

The 8 interlinked Millennium Development Goals to be achieved by 2015 and country commitments to the World Fit for Children fueled monitoring and measurement activities on child health and sparked the establishment of several global accountability initiatives that included regular quantitative progress assessments.^5^ The Countdown to 2015 initiative, for example, was launched in 2003 as a rallying cry to hold the world to account for progress toward Millennium Development Goal 4 on child survival.^11^ In 2005, Countdown published its first global progress report and pioneered a set of 1-page country profiles for the highest child mortality countries based on a standard template and the inclusion of indicators for which robust, comparable data were available. This template compiled all key data on child health ranging from demographics, intervention coverage, equity, nutrition, water and sanitation, and health policies and systems in 1 place and presented them through easy-to-grasp bar and line charts.^12^^–^^14^ Technical advisory groups, such as the Child Health Epidemiology Reference Group (now the Maternal and Child Health Epidemiology Estimation Group), were also formed to support measurement advancements in child health.^5^

Countdown was not alone in its production of statistical profiles to catalyze action. The Millennium Development Goal era ushered in a wave of similar data visualization tools produced by other maternal and child health-related initiatives, such as the Every Newborn Action Plan,^15^ Family Planning 2020 (now Family Planning 2030),^16^ and the African Leaders Malaria Alliance.^17^ United Nations agencies also began producing their own statistical snapshots to support country programming and monitoring and evaluation efforts (e.g., UNICEF's HIV^18^ and immunization^19^ statistical profiles, among others).

The propagation of data visualization tools on child health, including mobile-friendly applications and web-based data portals with interactive interfaces, has escalated during the Sustainable Development Goal era in part because of their popularity and advancements in technology, making them easier and cheaper to build.^20^ Data portals that enable users to generate graphical displays of child health data, for example, are now available for the Global Strategy for Women's, Children's, and Adolescents' Health (2016–2030)^21^ and the World Bank's Global Financing Facility for Women's, Children's and Adolescents' Health.^22^ Many universities, research institutions, and international nongovernmental organizations also publish data visualizations to promote advocacy and accountability (e.g., the Institute for Health Metrics,^23^ Save the Children's and UNICEF's co-produced Child Health Spotlights,^24^ University of Oxford's Our World in Data,^25^ and the Johns Hopkins University Lives Saved Tool^26^ among many others). Data collection platforms, such as the Demographic and Health Surveys, Multiple Indicator Cluster Surveys,^6^^,^^7^ and the DHIS2,^27^ provide applications for users to create graphics. In response to increased demands for data during the first years of the COVID-19 pandemic, numerous dashboards were created to help countries monitor and address the pandemic's impact on essential health services for women and children (e.g., WHO and UNICEF's pulse survey trackers^28^^,^^29^).

## THE NEED FOR STREAMLINING AND HARMONIZING STATISTICAL PROFILES ON CHILD HEALTH

The abundance of statistical dashboards on child health has helped put data into the hands of decision-makers and civil society actors and has made data more digestible and interpretable to nontechnical audiences. Their promotion through webinars and electronic communications has also helped keep children visible on the global landscape and further promoted the democratization of data (e.g., webinars hosted by the Health Data Collaborative and information shared through the Partnership for Maternal, Newborn, and Child Health monthly e-blasts). However, the sheer volume of data visualization tools has resulted in some discordance, redundancy, and frequent overlap in the information presented. There are many country profiles generated at the global level that include the same or similar child health-related indicators yet define or label them differently, use different data sources for them, update them at different time points, or display them in inconsistent ways, creating confusion. As an example, the Table presents variations in the display of care-seeking indicators for childhood illnesses on 3 global child health-related dashboards. These variations can result in confusion over the latest coverage values for these indicators, given differences in timelines for updating and how they should be interpreted and monitored. For example, a user may not be able to determine if care-seeking for symptoms of acute respiratory infection, as shown on the Countdown profile, is the same or a different indicator from care-seeking behaviors for pneumonia as presented on the Child Health Spotlight.

**TABLE.** Examples of Inconsistencies in Key Global Data Visualization Tools for Child Health and Well-Being, Examination of Care-Seeking for Childhood Illnesses Indicators

Global Initiative or Collaborating Institutions	Data Visualization Tool	Child Health Indicator Label on Tool	Graphical Display/Data Presentation and Metadata Documentation	Frequency of Updating
Countdown to 2030	Country profile for all low- and middle-income countries	Care-seeking for symptoms of acute respiratory infection	Bar chart showing trends Equiplot showing difference in coverage between poorest and richest quintiles Metadata including definition and data source available as an interactive hover function and in an annex	Annual, coinciding with updating of the UNICEF global databases
		Care-seeking for fever	No graphical presentation, latest national estimate and year presented	
Children in All Policies 2030	Child health and well-being dashboards for all countries organized by regions (World Health Organization, UNICEF, Sustainable Development Goal regions)	Care-seeking for fever	Traffic light graphical presentation (red, yellow, and green) Interactive hover function provides full metadata and classification as good progress, moderate progress, needs urgent attention, information provided on how classification categories were developed	Aim is annual, although cadence and timeline not firmly defined
UNICEF and Save the Children	Child Health Spotlights for 15 highest burden pneumonia countries	Care-seeking behavior for pneumonia	Key messages format, no graphics Reference to Global Action for Pneumonia and Diarrhea target National estimate Comparison between urban/rural and richest and poorest households Data source listed next to statistics, links to UNICEF and Save the Children website are provided at bottom of webpage for more information, metadata/annex on data sources and definitions not available	No stated commitment on regularity of updating
		Care-seeking behavior for diarrhea	Key messages format, no graphics Reference to Global Action on Pneumonia and Diarrhea target National estimate Comparison between urban/rural and richest and poorest households Data source listed next to statistics, links to UNICEF and Save the Children website provided at bottom of webpage for more information, metadata/annex on data sources and definitions not available	

## A WAY FORWARD: OPPORTUNITIES AND RECOMMENDATIONS FOR HARMONIZING DATA VISUALIZATIONS FOR CHILD HEALTH

In recognition of these problems and their contributory role, WHO and UNICEF began working closely together in recent years to improve coordination and streamlining of their data visualization tools on women's, children's, and adolescents' health. For example, through the Children in All Policies 2030 initiative, WHO and UNICEF led an extensive consultative process to create a simple dashboard in support of the Convention on the Rights of the Child.^30^ This dashboard was launched in May 2022 simultaneously on WHO, UNICEF, and Children in All Policies 2030 websites, and a plan was established to continue jointly developing and updating the back-end databases and the front-end data display. Both agencies are also putting procedures into place to ensure their websites are consistent in terms of data sources used, timing of updates, and how data on women's, children's, and adolescents' health are visualized.

To help users navigate and understand the complementarity of existing data visualization tools on women's, children's, and adolescents' health, UNICEF, in collaboration with WHO, created a webpage to compile key data visualization tools and links to other resources, including related topic-specific data visualization tools, global initiatives, journal articles, blogs, and reports.^31^ This webpage will need continuous curation to keep pace with new data visualization tools and the changing evidence base.

The following proposed set of good practices for producing data visualization tools, particularly for global monitoring, were developed by the WHO-UNICEF co-convened Child Health Accountability Tracking (CHAT) Technical Advisory Group.^32^ A function of CHAT is to help harmonize indicators and information on child health. This set was developed based on review of data visualization theory and experiences to date with the design and dissemination of child health and well-being dashboards, including the example of WHO and UNICEF working together on the Children in All Policies 2030 dashboards.^1^^,^^2^^,^^20^^,^^33^

1. Data visualization tools for global monitoring on child health should ideally be cocreated across organizations (including United Nations agencies and other institutions) to reduce duplication, increase their impact, ensure consistency in key messages, and ensure sustainability. Agencies should use their convening power to promote and facilitate coordination across key stakeholders on the development of data visualization tools if possible.
2. Donors should better align their requests for new data visualization tools on child health to reduce inefficiencies and the development of tools with limited use. Existing global and country mechanisms that adhere to the Paris Declaration on Aid Effectiveness and the Accra Agenda for Action^34^ can play a role in streamlining requests for new data visualizations (e.g., country platforms for donor coordination, Global Financing Facility Investors Group, and the United Nations H6+ Partnership). The Partnership for Maternal, Newborn, Child and Adolescent Health can also serve as a coordination mechanism.
3. The purpose and target audience of any new data visualization tool for child health should be defined at the outset to avoid duplication and to clarify its added value (Box 1).
4. Data visualization tools should be developed through an inclusive and consultative exercise. Some options include the creation of an advisory group composed of a diverse representation of actors, a wide consultative process done through an open web-based platform, or a tailored delphi-like process that solicits inputs from all key stakeholders. Country actors and civil society organizations should be engaged throughout the entire development process to ensure any new data visualization tools on child health meet their decision-making or advocacy-related needs. Technical and data visualization experts should also be involved in all steps so that the tool is based on the latest scientific evidence and graphical techniques.
5. The development of a data visualization tool should be iterative, with a lead institution or individual managing and building consensus throughout the process (Box 2). This consensus-building process involves engaging with all partners identified in principle 4.
6. Indicators selected for inclusion on data visualization tools should ideally be based on standard definitions and recommended data sources, as well as be readily available through existing data collection platforms. They should be as consistent as possible with indicator recommendations from the following 3 technical advisory groups established to promote indicator harmonization and standardization: Mother and Newborn Information for Tracking Outcomes and Results,^35^ CHAT,^36^ and the Global Action for Measurement of Adolescent Health.^37^

BOX 1 Checklist for Defining the Purpose of a Data Visualization Tool for Child Health and Well-Being

- Identify the target audience (e.g., local decision-makers, civil society organizations, health care professionals, policymakers, donors or other development partners, regional organizations, etc.).
- Specify what need the tool will fulfill (e.g., will it provide information on a specific topic in child health, or will it enable progress assessments across a set of child health issues, etc.).
- Determine if the tool will add value. This decision may involve reviewing existing tools and assessing whether a new tool will provide needed and actionable complementary information. The continuation of the mapping work started by UNICEF and the World Health Organization on data visualization tools for women's, children's, and adolescents' health, with additional information on the intended purpose and target audience for each tool, is important for facilitating this step.
- Ensure the tool's use will support an integrated, holistic approach to child health. For topic or thematic-specific tools, efforts should be made to determine how they can be used in combination with other more comprehensive tools to avoid continued fragmentation in child health. Data visualization tools specific to childhood immunization, for example, could be expanded to include health systems indicators or links to other relevant tools could be provided (e.g., data visualizations that place immunization services in the context of other child health interventions and/or information on health system characteristics).
- Ensure the format and technical sophistication of the graphics and accompanying materials are appropriate for the target audience.
- Balance the value of adding features like interactivity to improve the tool's uptake with their costs.
- Consider specific events or activities where the tool could be used that are in line with the tool's purpose. For example, statistical dashboards on child health could be included in annual reviews of national health plans or used to stimulate dialogue in convenings of multistakeholder country platforms where they exist.

BOX 2 Checklist on Developing a Dashboard Through an Inclusive, Consultative Process

Through discussions facilitated by a lead institution or individual, decisions should be reached on the following:

- Agreement on the name and purpose of the tool, as well as assurance that the tool adds value and is not duplicative
- Selection of the indicators, including types (e.g., input, output/process, outcome, impact, and contextual factors) and definitions (using standard definitions, data sources, and available data collection mechanisms as possible)
- How the back-end database is compiled, including which data sources are used and data quality review processes, as well as the process for finalizing data sharing agreements for all data that are not from publicly available sources
- Layout of the tool template (e.g., number of pages and whether it will be standard or customized for each country)
- How each indicator will be visualized (e.g., table, chart, or map) and organized on the template (e.g., where it will be placed in relation to other indicators)
- Inclusion on the template of any goals, targets, key messages, or icons for performance reporting (e.g., traffic light color coding or the use of arrows)
- Inclusion of interactive features, language options, and different versions depending upon bandwidth and other requirements of intended users (e.g., application on phones and other digital devices, print versions where Internet is not accessible, etc.)
- Development of accompanying materials (e.g., annexes with metadata, video tutorials or guidance materials on how to use the tool and interpret the data, data downloads, downloadable data visualization tools or specific charts, and short reports or brochures with key messages)
- Inclusion of logos or other ways of acknowledging partners involved in the development and/or dissemination of the tool
- Roles and responsibilities for building and maintaining the tool and the back-end databases to ensure sustainability
- Dissemination strategy, including where the tool will be hosted online and how it can be downloaded
- Frequency of updating the front- and back-end of the tool, inclusion of a feedback mechanism for real-time inputs from users, and analytics for tracking use
- Dissemination plans and venues or events where the tool could be used to support discussions and decision-making
- Resource mobilization for the development, updating, maintenance, and dissemination of the tool

Although these good practices are geared for the development of global, regional, or national level tools that allow for comparisons across countries and time, most are applicable to the development of subnational data visualization tools based on local data sources as well. If these practices are followed, the usefulness and quality of data visualization tools that can spur action and drive accountability for child health and well-being could be substantially improved.

## Notes

**Peer Reviewed**

**First published online:** December 8, 2023.
